# Using Community-Based Participatory Research to Develop Care Recommendations for People Aging With HIV

**DOI:** 10.1093/geroni/igad107

**Published:** 2023-09-25

**Authors:** Maria T Brown, John Wikiera, Marz Albarran, Angie Partap, Courtney Ahmed, Victoria Brock, Sheriden Beard, Eugenia L Siegler

**Affiliations:** School of Social Work, Syracuse University, Syracuse, New York, USA; New York State Department of Health AIDS Institute, Albany, New York, USA; Albany Damien Center, Albany, New York, USA; Stony Brook Medical University, Stony Brook, New York, USA; New York State Department of Health AIDS Institute, Albany, New York, USA; Cicatelli Associates Inc., New York, New York, USA; New York State Department of Health AIDS Institute, Albany, New York, USA; The Center on Aging, Weill Cornell Medicine, New York, New York, USA

**Keywords:** Chronic illness, Community-based participatory research, HIV/AIDS, HIV aging, Quality of care

## Abstract

**Background and Objectives:**

Over 50% of New Yorkers living with human immunodeficiency virus (HIV) are 50 years old or older, and the emotional and physical consequences of being a long-term survivor are significant. This study aimed to identify the practical needs of long-term survivors and older people with HIV (consumers) in New York State and develop recommendations addressing those needs.

**Research Design and Methods:**

The HIV + Aging/LTS/Perinatally Diagnosed Subcommittee of the Consumer Advisory and Quality Advisory committees in the New York State AIDS Institute used community-based participatory research (CBPR) methods to design a statewide survey about the care needs of consumers in New York State. This survey, open to consumers, clinicians, and supportive services providers, was launched in June 2021 using Qualtrics. Participants provided demographic data and chose the 3 most important barriers and recommendations from each of 10 categories of issues affecting health care and supportive services. Consumers provided information about their HIV diagnosis and other health conditions. Responses were characterized using basic descriptive statistics.

**Results:**

Participants included 124 consumers from 26 counties, 20 clinicians, and 24 supportive service providers. Among consumers, 67% were cisgender men, 27% were African American, and 65% were both long-term survivors and older people with HIV. On average consumers had been diagnosed with HIV for 27 years. Participants were concerned with clinical care coordination, housing needs, cultural representation in mental health services, and financial support of consumers.

**Discussion and Implications:**

CBPR is an effective approach to developing consumer-generated recommendations to improve HIV care for long-term survivors and older people with HIV. Town hall formats informed survey design, enabled broad coverage of topics, and ensured that focus remained on priorities most important to consumers. The first quality initiative arising from the study was a routine screening of long-term survivors of HIV to identify functional decline and enhance referral pathways and care linkages.

**Translational Significance:** Despite the changing epidemiology of the human immunodeficiency virus (HIV) population, research has not kept pace, and care for long-term survivors and older people with HIV is not standardized. The current study demonstrated it is possible to create an academic-community statewide partnership to initiate community-based participatory research projects, provide a mechanism for using research findings to advocate for change and improve the care of long-term survivors and older people with HIV. The New York State AIDS Institute has led the first quality improvement initiative based on the survey’s recommendations and continues to review these recommendations to inform quality initiatives and improve clinical care for long-term survivors and older people with HIV.

## HIV and Long-Term Survivorship

First recognized in the United States in 1981, what would be named acquired immunodeficiency syndrome (AIDS) was nearly always fatal and had a major impact on public health (Centers for Disease Control, 1981). Among people 25–44 years of age in 1989, human immunodeficiency virus (HIV) infection/AIDS was responsible for 14% of deaths among men and 4% of deaths among women (Centers for Disease Control, 1991). With the advent of effective antiretroviral therapy for HIV in the 1990s, mortality rates have declined dramatically; although these rates remain higher than in the general population (Edwards et al., 2021), most people are now able to live with HIV as a chronic disease. As a result of declining new infections and markedly improved lifespan for those with HIV, the population with HIV is aging.

By the end of 2019, 56% of people living with HIV in New York State were 50+ (Bureau of HIV/AIDS Epidemiology, 2020). Because people with a suppressed viral load can no longer infect others (Undetectable = Untransmittable), the goal of ending the epidemic is achievable and a high priority in New York State and globally (Centers for Disease Control, 2021; Morne et al., 2020; The Joint United Nations Programme on HIV/AIDS [UNAIDS], 2015, 2018). In the absence of a cure, the emotional and physical consequences of being a long-term survivor are significant. Understanding how to meet their needs is essential to improving their quality of life.

People with HIV appear to experience advanced aging, with a biological age up to 15 years older than their chronological age (De Francesco et al., 2019). They are at higher risk for multimorbidity, depression, loneliness, and geriatric syndromes such as frailty (Do et al., 2014; Falutz et al., 2018, 2021; Greene et al., 2018; Guaraldi, 2018; Weinstein et al., 2021). Food insecurity and lack of access to affordable housing are significant concerns (Aidala et al., 2016; Anema et al., 2011; Freiria, Arikawa, Van Horn, Corona & Wright, 2022). The Corona Virus Immune Disease-2019 (COVID-19) pandemic has increased these biopsychosocial stressors (Shiau et al., 2020). Another group of long-term survivors who have received less attention are those who acquired HIV perinatally. With antiretroviral therapy, perinatal transmission is now extremely rare in the United States. Nonetheless, many of the perinatally diagnosed are now transitioning care from pediatricians to adult medicine providers and are at high risk of dropping out of care (Flynn & Abrams, 2019). Now in their 20s and 30s, they may be experiencing advanced aging (Dalzini et al., 2020) and many of the same comorbidities as their older counterparts (Centers for Disease Control, 2018; Flynn & Abrams, 2019).

### Community-Based Participatory Research

Community-based research is a collaborative approach to public health research in which community, public health, and academic researcher partners are equitably engaged in all aspects of the research. Each partner offers unique skills and strengths and shares responsibility for enhancing the team’s understanding of the phenomenon and community being studied. All partners work together to integrate knowledge produced by the research with action to improve community members’ health and well-being (Israel et al., 1998, p. 177). Community-based participatory research (CBPR) is one approach to community-based research that ensures community members have full and equal partnership in all aspects of the research (Rhodes et al., 2011). CBPR addresses health equity concerns by engaging community partners’ insights into structural barriers, environmental factors, cultural practices, and health behaviors that can inform traditional health promotion, prevention, and clinical care (Israel et al., 2005; Senteio et al., 2021). CBPR may be particularly salient for research intended to identify the needs of marginalized communities, like people of color and people living with HIV (Weinstein et al., 2021).

#### CBPR and marginalized communities

Community-based participatory research methods have been proven to ensure a broader representation of marginalized groups living with HIV (Abelsohn et al., 2015). Previous research involving gay and bisexual men who have sex with men and transgender women of color in a community with high incidence rates of sexually transmitted infections and HIV found that CBPR, by involving stakeholders, could engage community members, identify needs and priorities, and inform the development of interventions tailored to their communities’ needs (Mann-Jackson et al., 2021). Past CBPR studies of communities living with HIV have identified barriers to participation in research, including lack of researcher respect for community-based organizations and inadequate attention from researchers to the need to build trust (Safo et al., 2016). Members of the HIV-positive community have reported feeling like “token” members of the research team, feeling disempowered by poor communication from the researchers, and perceiving a power imbalance because of researchers’ preconceived beliefs about the HIV-positive community.

The current project, rather than being initiated by the researcher to achieve their research goals, was initiated by members of the HIV-positive community in New York State, to bring to light quality of care issues being reported by members of that community. The community members who initiated this study were volunteers in the advisory committee structure of the New York State AIDS Institute.

### The Current Study

Despite the changing epidemiology of HIV, clinical research has not kept pace, and clinical care for long-term survivors and older people with HIV is not standardized (Davis et al., 2022; Levin & Montano, 2019). Understanding the most pressing issues and developing best practices for addressing them requires close collaboration with people with HIV (Karris, Dubé & Moore, 2020). Such collaboration can be achieved through CBPR, where consumers and academics work together in every step of the research process (Rhodes, Malow & Jolly, 2010). In New York State, a consumer advocacy group with connections to HIV clinicians and social science researchers organized a consumer-academic collaboration to design and administer a statewide web-based survey. This survey would address the research question: What are the unmet clinical and social needs, and how can we meet those needs, of long-term survivors and older people with HIV in New York State? With this survey, the CBPR team queried consumers and providers about barriers to quality care and proposed solutions to those barriers, for development into quality improvement initiatives. This article describes the CBPR process through which this research was conducted, describes the results of the survey, and concludes with a discussion of the next steps being taken in New York State to address quality of care issues across the state, including the development of quality improvement initiatives and recommendations to improve quality of care for long-term survivors and older people with HIV (Solomon et al., 2016).

## Method

### New York State AIDS Institute

The New York State AIDS Institute was legislatively established within the New York State Department of Health Office of Public Health in 1983 through Public Health Law Article 27-E, as part of the state’s health care response and comprehensive public health approach to HIV/AIDS (AIDS Institute, 2021). The AIDS Institute’s Office of the Medical Director sponsors multiple initiatives including the HIV Quality of Care Program. Quality of Care initiatives for adult consumers with HIV include the Consumer Advisory Committee and Quality Advisory (clinicians) Committee.

These stakeholder committees meet independently and jointly to represent the diverse communities and regions of New York State affected by the HIV epidemic and to advise on the development, implementation, and refinement of the Quality of Care program (Coren et al., 2021; Department of Health, 2022). In March 2020, the Consumer Advisory and Quality Advisory committees voted to create a joint subcommittee on HIV + Aging, Long-term Survivors, and Perinatally Diagnosed (HALP), with representation among the co-chairs for long-term survivors and older people with HIV, the perinatally diagnosed, women of color, and clinicians. The co-chairs subsequently invited the researcher to serve as a co-chair for the purpose of conducting this study. Although AIDS Institute staff assisted the subcommittee in this research, the AIDS Institute did not conduct the study.

To identify the priorities of consumers across New York State, the HALP subcommittee hosted two virtual town halls in August 2020 to hear consumers’ concerns about health care and supportive services for long-term survivors and older people with HIV. Community members across the state were invited to engage the perspectives of consumers, clinicians, and supportive service providers. The first town hall focused on quality of care issues encountered in health care or supportive services, and the second town hall explored quality of care issues encountered in supportive services. During these town halls, small group discussions were facilitated by one HALP co-chair and one note-taker. Participants in the small group discussions were asked about problems they had encountered in health care and supportive services and to suggest recommendations to address these problems. The researcher observed these town halls but did not actively participate in them.

### Research Design and Implementation

**Figure 1.**
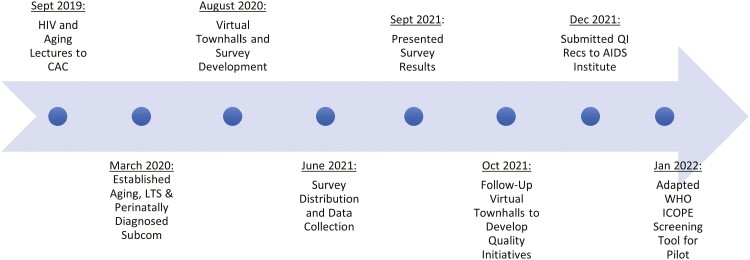
HIV+ Aging and Long-Term Survivorship Study Timeline. CAC = Consumer Advisory Committee; QI = quality initiative; ICOPE = integrated care for older people; Recs = recommendations; Subcom = subcommittee; WHO = World Health Organization.

The research team was comprised of HALP co-chairs (three consumers, one clinician, and the researcher) and AIDS Institute program staff. This study was initiated as a community-based project, by consumers, to address concerns they had identified through personal experience. We used CBPR methods in the planning, research design, research implementation, and action phases of the project (see Figure 1) to foreground consumer experiences in the design and implementation of the study, to engage consumer perspectives to guide the interpretation and reporting of study findings, and to support consumers as they developed action plans based on those finding (Brizay et al., 2015). In the planning phase, all members of the research team collaborated with the researcher to identify the research question, develop the research plan including the informed consent process, and obtain ethical approval for the research.

In the research design phase, the research team used deidentified notes from the aforementioned town halls to create a statewide survey of consumers, clinicians, and supportive service providers, using Qualtrics (see Supplementary Section B). In virtual meetings over the course of 9 months, the team identified various categories of issues affecting quality of care and organized town hall comments into lists of barriers to quality care and recommendations to address those barriers for each category. The categories were consolidated into 10 broad categories: five in health care and five in supportive services. All team members pretested the survey prior to distribution. Additional AIDS Institute staff were recruited to pretest the survey, prioritize questions, and optimize survey length. This project was approved by the Syracuse University Institutional Review Board.

In the research implementation phase, community members of the research team recruited participants by distributing survey invitations via an anonymous survey link by email and social media through the Consumer Advisory and Quality Advisory committees and other state-wide consumer groups, for distribution to their networks. The survey was opened in June and closed the first week of August 2021 and was open to all adult New York residents with HIV who were either 50 and older or identified as long-term survivors of any age, and those who provided health care or supportive services for them. The survey did not define long-term survivors, as subcommittee members felt strongly that it was up to the individual consumer to self-identify as such.

Survey participants were asked for basic demographic data (including whether they were a person living with HIV, a clinician, or a supportive services provider), and consumers were asked about their county of residence, health status, and comorbidities. For each of the 10 categories, a list of barriers and recommendations was provided, based on the town hall data. All survey participants were asked to choose the top three barriers and recommendations from the lists in each category. There was an option in each list for participants to choose “other” to add new items.

In the action phase of the project in the fall of 2021, the research team evaluated the survey findings and conducted town halls with Consumer Advisory and Quality Advisory committee members to develop quality initiatives based on recommendations from the survey. At the time of writing, the HALP co-chairs continue working with the advisory committees and AIDS Institute staff to plan interventions based on these quality initiatives. The first set of recommendations was focused on statewide screening of the functional capacity of long-term survivors and older people with HIV.

### Analysis Plan

The current descriptive study applied simple univariate analysis to determine the identifying characteristics of participants. We applied bivariate analyses to evaluate the health concerns of long-term survivors and older people with HIV. We compared the health concerns of consumers who identified as both long-term survivors and older people with HIV (*n* = 81) versus those who identified as being only long-term survivors or older people with HIV (*n* = 43), using Chi-square tests of significance. Finally, we conducted content analysis to identify the top three (or four if two items appear with the same frequency) barriers and recommendations most frequently selected by consumers in each category. The current study focuses on the concerns of consumers, as fewer clinicians and supportive services providers responded to the survey.

## Results

### Sample Description

As shown in Table 1, survey participants included 124 consumers from 26 New York counties, 20 clinicians, and 24 social service providers. Two-thirds of participants (67%) were cisgender men, 27% were African American, and 65% identified as both long-term survivors and older people with HIV. On average, consumers were 58 years old (*SD* = 9.24, range 30–85), had been living with their HIV+ diagnosis for 27 years, and reported four additional chronic health conditions, most commonly high cholesterol (40.3%), high blood pressure (36.3%), depression (39.5%), anxiety (35.5%), being overweight (35.5%), and taking medications for pain (28.2%). Two-thirds of consumers were living in cities, and 8% in rural areas.

**Table 1.** Sample Characteristics, HIV Aging Survey, 2021

Characteristics	Consumers (*n* = 124)	Clinicians/providers (*n* = 44)
Age, *M* (*SD*)	58.22 (9.24)	50.48 (13.75)
Range	30–85	21–82
Gender identity (%)		
Male	67.2%	31.0%
Female	31.9%	66.7%
Trans/nonbinary/gender non-conforming	0.8%	2.4%
Race/ethnicity (%)		
White or Caucasian	57.3%	61.4%
Black, African American, or Afro-Caribbean	28.2%	18.2%
Hispanic or Latinx	11.3%	15.9%
South Asian	0.0%	2.3%
Southeast Asian	0.6%	2.3%
Middle Eastern or North African	0.8%	0.0%
American-Indian or Alaska Native	0.8%	2.3%
Other race or origin	3.2%	0.0%
Years living with HIV diagnosis, *M* (*SD*)	26.76 (8.07)	
Range	3–39	
Identity as HIV survivor (%)		
Both Age 50 or older and long-term survivor	65.3%	
Age 50 or older only	14.5%	
Long-term survivor only	13.7%	
Neither	1.6%	
Missing	4.9%	
Relationship status (%)		
Married or living with partner	37.8%	
Single	47.1%	
Other status	15.1%	
Living arrangements (%)		
Living alone	45.8%	
Living with spouse or partner	38.1%	
Other arrangement	16.1%	
Location of residence (%)		
City	65.8%	
Suburb	26.5%	
Rural/other	7.7%	
Years treating HIV patients, *M* (*SD*)		15.95 (9.86)
Range		0–35
Location of practice (%)		
City		81.8%
Suburb		9.1%
Rural/other		9.1%

Consumers who identified as both long-term survivors and older people with HIV reported having 4.8 additional chronic conditions on average, compared to an average of 2.7 for those who identified as being in either group (Supplementary Table 1). Consumers who identified as both long-term survivors and older people with HIV were significantly more likely than consumers who identified as long-term survivors only, older people with HIV only, or neither long-term survivors nor older people with HIV, to report having a history of cancer (23.5% vs 4.7%, *p* < .05), diabetes (12.3% vs 2.3%, *p* < .01), heart disease (18.5% vs 4.7%, *p* < .05), high cholesterol (46.9% vs 27.9%, *p* < .05), history of hepatitis (28.4% vs 9.3%, *p* < .05), hypertension (46.9% vs 16.4%, *p* < .001), or lipodystrophy (29.6% vs 9.3%, *p* < .001). They were also significantly more likely to report being overweight (43.2% vs 20.9%, *p* < .001) and taking medications for pain (34.6% vs 16.3%, *p* < .05). Supplementary Table 2 shows that our sample, relative to the total HIV+ population in New York State, oversampled the concerns of HIV+ New Yorkers outside of the New York City region. This distribution ensured that, while our sample represented only 1.1% of New Yorkers living with HIV, it did represent the needs of a diversity of regions across the state.

A total of 20 clinicians and 24 supportive services providers also responded to the survey. This group was 50 years old on average (*SD* = 13.75, range 21–82). The majority were White (60%) and female participants (67%). The majority of clinicians and providers were practicing in cities (81%). The subset (*n* = 20) of clinicians/providers who responded to the questions on their practice had been working with HIV+ patients for an average of 16 years (*SD* 9.79), and the majority were working in academic practice (60%) and exclusively with HIV+ patients (60%).

The most common barriers and recommendations selected by consumers are shown in Table 2, ranked by the percentage of consumers who endorsed each item. Long-term survivors and older people with HIV were most concerned about clinical and financial needs, particularly coordination of clinical care, unmet housing needs, access to mental health services, barriers related to COVID-19, cultural representation in mental health services, and financial support of long-term survivors and older people with HIV. The recommendations most frequently endorsed by consumers addressed housing needs, financial support for long-term survivors and older people with HIV, telehealth, clinical care coordination, mental health and social support, and barriers related to COVID-19.

**Table 2.** Most-Endorsed Barriers and Recommendations to Improve Quality of Care, by Percentage of Consumers, HIV Aging Survey, 2021

Barrier	%	Recommendation	%
Clinical care coordination			
Lack of care coordination across different departments and specialties	20.87	Integrate HIV care with other specialties so people living with HIV have different needs met in one place	22.05
Lack of support to help people living with HIV manage care	16.99	Increase funding for clinics and facilities in underserved and understaffed areas	21.29
Not enough HIV primary and specialty care providers in upstate NY and rural areas	16.50	Provide clinicians with relevant experience to help older people living with HIV and long-term survivors manage their complex care needs	20.15
		CPWs should be included in initial visit with provider and also assist people living with HIV in managing medications, etc.	18.25
Telehealth/Telemedicine			
Lack of knowledge or comfort with evolving technology	17.37	Provide subsidies so people living with HIV can afford current technologies for telehealth	22.69
People living with HIV cannot afford to access technology (i.e., phone plan, internet plan, Wi-Fi, etc.)	17.37	Pay attention to the psychological impact of transitioning to or starting care with telehealth	23.11
Telehealth makes it harder for people living with HIV (especially newly diagnosed) to discuss their medical issues and connect with their clinician	17.37	Mandate annual in-person visits to ensure that people living with HIV’s needs are being met	23.11
Medication and pharmacy services			
Up to pharmacists to catch potential drug interactions when the primary care doctor is not the sole prescriber of medication	25.50	More education and trainings for pharmacy staff on cultural sensitivity, confidentiality, and insurance coverage	21.33
Pharmacists need training to understand HIV from the consumer’s point of view	20.00	Give people living with HIV a day or two of medications before leaving the ER	17.78
Medication delivery services are not always reliable or available at all pharmacies especially during the COVID-19 pandemic	18.50	Advertise AIDS Institute HIV medication assistance telephone number (and push to include ALL medications an HIV+ person is currently taking)	17.33
**Aging and Long-Term Care**			
People living with HIV have multiple health conditions and aging-related complications occurring with HIV that are not acknowledge or addressed	18.95	Provide assisted living and non-assisted living options for older people living with HIV	19.15
Long-term care facilities are not equipped to take care of people living with HIV and sometimes reject them because of expensive HIV medications	12.90	Educate people living with HIV and providers on the effect of HIV/AIDS on aging and comorbidities for people living with HIV of different ages	15.96
People living with HIV face stigma and homophobia in assisted living facilities	12.90	Aging people living with HIV and long-term survivors need better care coordination between providers, social workers, and mental health services	9.22
		Standardize infrastructure and policies so long-term care facilities can provide better services for people living with HIV and people with mobility challenges	9.22
Mental health and social support			
Lack of access to self-care services, alternative therapies, and wellness centres	22.17	Hire counsellors and mental health professionals that represent communities they serve	21.74
Lack of therapists/counsellors that are representative of clients (share the same race, gender, age, or cultural background)	20.00	Providers should routinely ask people living with HIV about their mental health	21.34
Not enough coverage provided for mental health through Medicaid or other insurance plans	20.00	Mental health programs that are multilingual, culturally inclusive, and culturally sensitive	15.81
Barriers related to COVID-19			
Not being able to see people or attend support groups in person due to COVID-19	27.35	CPWs should make regular check-in calls to people living with HIV who are isolated during COVID-19	23.33
Not enough mental health providers to help people living with HIV cope with the added trauma of COVID-19, such as isolation and depression	22.22	Notify people living with HIV when in-person appointments are available, rather than leaving the burden on people living with HIV to keep checking	22.50
During the pandemic, it is easy to isolate, drop out of care, and not be held accountable	19.66	Make one-on-one counselling widely available through telehealth during and after COVID-19	20.00
Health Equity and Stigma			
People living with HIV face stigma in both HIV and non-HIV specialties (e.g., dentistry) based on their race, immigration status, sex work, and/or substance use	16.73	Community partnerships should provide more regular training for all health care staff on trauma-informed care, cultural competency, and HIV services	18.05
Lack of HIV research done on women, older adults, and heterosexual people	13.06	Diverse people living with HIV (age, modes of transmission, etc.) and demographic representation among community boards, clinic staff, and supportive services staff that people living with HIV can identify with	11.28
Lack of training in trauma-informed care, cultural sensitivity, and HIV services for all health care staff	12.24	Have CPWs on staff to help people living with HIV communicate with their doctor and ask questions	10.15
Transportation and food			
Lack of public transportation (e.g., buses, taxies, subways, etc.) that is affordable, available, and accessible to all no matter their age, income, or insurance status	19.46	Provide more affordable and accessible transportation options for aging and mobility-challenged people living with HIV	21.26
It is difficult for people living alone who can’t cook on their own to access healthy foods	15.84	Provide food delivery options for aging and mobility-challenged people living with HIV	19.29
Pantries have limits and may not have food for people with specific nutritional needs	14.93	Use CPWs to educate people living with HIV on resources including food vouchers and local food banks	14.17
Housing			
There are long waiting lists for housing	28.45	Build more housing and make more housing available to low-income populations	32.02
Lack of safe, accessible, and affordable senior housing	26.36	Agencies that receive housing assistance funds should include consumer input and clarify their definitions of affordable housing	24.51
Lack of attention and care for homeless population living with HIV	14.23	Make homelessness agencies aware of HIV-related needs	17.79
Financial support			
Even with insurance, copays are too high for emergency or urgent care	20.24	More funded programs to help people living with HIV pay for housing, food, and transportation	25.19
People living with HIV do not know enough about Social Security and Disability (SSI and SSDI) benefits	19.84	More leeway/unlimited extra income for people living with HIV over 50 who have SSI/SSDI	15.79
An increase in income reduces SSI/SSDI benefits and coverage for Medicaid, copays, medical visits, housing benefits	14.57	Better advertising of services to help people living with HIV obtain high quality health insurance or help pay for medications (for example, ADAP)	12.78

*Note*: ADAP = AIDS Drug Assistance Program; CPW = Certified Peer Worker; SSI = Supplemental Security Income; SSDI = Social Security Disability Insurance; NY = New York.

Clinicians and supportive services providers frequently endorsed the same barriers and recommendations as consumers. For example, all three groups ranked the lack of care coordination across different departments and specialties as the most important barrier to clinical care coordination. Similarly, they endorsed the same three barriers related to COVID-19, although in a different order; however, their selections in the health equity and stigma category were very different.

## Discussion

This study was developed at the initiation of and designed in collaboration with long-term survivors and older people with HIV from across New York State. We collected information about barriers to quality care encountered by older people with HIV and long-term survivors and recommendations on how to address those barriers. Participants identified barriers and made recommendations in 10 different areas. Specifically, they cited a need for better coordination of care, particularly for older people with HIV and/or long-term survivors and with multimorbidity and conditions associated with advanced aging, unmet needs for safe and affordable housing, and the financial challenges of living with HIV.

### Implications for Practice

As part of the ongoing action phase of this project, survey results were reported to the membership of the Consumer Advisory and Quality Advisory committees, whose members then began work on establishing and prioritizing potential quality improvement initiatives for consideration by the AIDS Institute. The first recommendation proposed to the AIDS Institute was one that committee members determined would be most actionable by the Institute and would improve clinical care for the largest number of long-term survivors, the perinatally diagnosed, and older people with HIV. This recommendation, to promote routine functional and mental health screenings of adult long-term survivors and older people with HIV, arose from prioritizing the barrier of a lack of care coordination in HIV care. The most endorsed recommendation among consumers that would address this barrier was to provide clinicians with relevant experience to help long-term survivors and older people with HIV to manage their complex care needs. The first step in providing this experience was to develop a comprehensive screening tool and guidelines for using that tool.

The HALP subcommittee collaborated with AIDS Institute staff on a statewide quality improvement initiative that provided a screening tool, workflow, and reporting template to clinical sites. The screening tool was a modified version of a global screen provided by the World Health Organization’s Integrated Care for Older People (ICOPE) Guidance (World Health Organization, 2021). HALP co-chairs and AIDS Institute program officers slightly modified the ICOPE screen to address concerns related to long-term survivorship and aging with HIV (AIDS Institute, 2023a).

The modified ICOPE screen and workflow were presented to the Consumer Advisory and Quality Advisory committees for feedback, approved by the AIDS Institute, and piloted as an AIDS Institute-sponsored quality improvement initiative to test the feasibility and value of administering the screen to long-term survivors, including the perinatally diagnosed, and older people with HIV by staff or certified peer workers at clinical sites; there is also an option to administer the screen via telehealth. The quality improvement pilot study testing the feasibility of the screening process will be described in detail in a future report.

Based on the success of this pilot, the Medical Care Criteria Committee of the AIDS Institute has included this modified ICOPE screening tool in their guidance on geriatric screening and assessment of older patients with HIV (AIDS Institute, 2023b). After the completion of this first quality improvement pilot, the HALP subcommittee chairs began the process of working with the Consumer Advisory and Quality Advisory committees to identify the next recommendation for development into a quality improvement initiative. Each new recommendation and proposal to the AIDS Institute is expected to follow a similar process.

### Strengths and Limitations

This study had several strengths resulting from the use of CBPR methods. First, the study arose from the interests of consumers in the community who engaged the researcher to assist them in implementing the study. Second, the inclusion of long-term survivors, the perinatally diagnosed, and older people with HIV on the research team ensured that consumer needs were prioritized in every phase of the research process, including the survey design. This prioritization of consumer needs was further enhanced by including consumers in the virtual town hall meetings that HALP conducted to identify barriers and recommendations for inclusion in the survey. The inclusion of clinicians and supportive services providers also enhanced our ability to include their perspectives on unmet consumer needs in the survey. Third, the participation of community members, clinicians, and supportive services providers in recruitment led to a greater representation of minority populations than usually found in traditional, researcher-driven research. As a result, 44% of our sample identified as a member of one or more racial/ethnic minority groups.

Fourth, because this study was conducted during the COVID-19 pandemic, we relied on electronic recruitment and data collection, which supported a broader reach across the state, resulting in more representative distribution of responses from all regions of the state (Nguyen, Christensen, Taylor & Brown, 2020). Finally, the inclusion of consumers in the research team, and of the Consumer Advisory and Quality Advisory committees in the development of quality improvement initiatives, such as the modified ICOPE instrument and guidelines, ensures that the recommendations developed by the HALP address the needs identified in the survey. Their inclusion also confirms the validity of these concerns and the appropriateness of the recommendation, increasing the likelihood that the AIDS Institute will act on our recommendations (Solomon et al., 2016).

These findings should be considered in light of several limitations in our study. First, the necessary reliance on electronic recruitment and data collection during the COVID-19 pandemic, which would typically occur in the community through HIV clinics, supportive service agencies, and consumer advocacy meetings, inherently excluded individuals who did not have access to internet technology for reasons of income or infrastructure (Nguyen, Christensen, Taylor & Brown, 2020). As such, our data likely underrepresents New Yorkers living with HIV in rural areas, who are of lower income, or of lower education or literacy. Therefore, our findings related to telehealth and telemedicine may not adequately reflect the challenges of accessing quality care that patients living with HIV were experiencing during the pandemic.

Second, this survey was only distributed in English, which inherently excluded persons living with HIV who did not read or write in English, and therefore underrepresented immigrants and non-English speaking populations. Third, our team experienced difficulties reaching traditionally underrepresented populations, including persons living with HIV who were younger, perinatally diagnosed, trans, or nonbinary; therefore, our results do not offer any insights specific to their experiences. The lack of participation from these traditionally underrepresented populations could be due in part to the social stigma of living with HIV, compounded by the social stigma of being a member of these communities. This stigma may inhibit people from sharing their experiences or participating in research if they are not familiar with the researchers or the organizations involved (Logie et al., 2014).

Despite these limitations, the HALP subcommittee has been successful in collecting survey data from a representative sample of New Yorkers living with HIV and in using survey results to promote changes to clinical care for long-term survivors and older people with HIV in New York State. The first of these changes, as described, was the implementation of the modified ICOPE screening tool to identify potential declines in the functional capacity of long-term survivors, the perinatally diagnosed, and older people with HIV, and to enhance referral pathways, linkages to care, and the overall quality of care of this population in participating organizations. In addition, the AIDS Institute Quality of Care Program offered quality improvement training and technical assistance, so that participating organizations could use the screening tool as a mechanism for identifying clinical processes that can be improved to enhance the quality of clinical care for long-term survivors and older people with HIV.

The HALP subcommittee worked with the AIDS Institute to oversee this pilot project, to report the results of the pilot to the Consumer Advisory and Quality Advisory committees, and to incorporate the resulting recommendation in the AIDS Institute’s guidance for addressing the needs of older people with HIV across the state. As an ongoing effort, the HALP subcommittee continues to meet to review other recommendations derived from the survey results and to develop more recommendations for presentation to the AIDS Institute.

## Conclusion

This study’s results revealed that long-term survivors and older adults with HIV need improved access to health care and social and financial support to address the challenges they face living with HIV in New York State. The research process demonstrated it is possible to create an academic-community statewide partnership to initiate community-based participatory research projects and to provide a mechanism for using research findings to advocate for change, and that long-term survivors, the perinatally diagnosed, and older people with HIV can collaborate with the AIDS Institute to identify problems and develop solutions to enhance their care. Although this project was realized by consumer populations in New York State, similar processes can be implemented by consumers in other communities. Furthermore, the process of creating, analyzing, and publicizing the CBPR project has motivated survey participants to advocate for specific changes recommended by their peers to address identified barriers to quality care. Based on the success the HALP subcommittee and the Consumer Advisory and Quality Advisory committees have had in bringing the first recommendation from the survey results into practice across the state, we anticipate that their advocacy using these survey results will lead to additional improvements in clinical care and supportive services for long-term survivors and older people with HIV in New York State.

## Supplementary Material

igad107_suppl_Supplementary_Tables_1-2
