# Supplementary material for: Using Community-Based Participatory Research to Develop Care Recommendations for People Aging With HIV
Source: Innov Aging. 2023 Sep 25;7(8):igad107. doi: 10.1093/geroni/igad107 (PMC10629937; doi:10.1093/geroni/igad107)
Supplement: igad107_suppl_Supplementary_Tables_1-2 [file igad107_suppl_supplementary_tables_1-2.docx]

***Innovation in Aging* Online Supplementary Material:** Maria Teresa Brown, John Wikiera, Marz Albarran, Angie Partap, Courtney Ahmed, Victoria Brock, Sheriden Beard, Eugenia L. Siegler. Using Community-Based Participatory Research to Develop Care Recommendations for People Aging with HIV

**Section A: Supplementary Tables**

Table S1. Chronic Conditions Reported, Comparing Both Age 50+ and Long-Term Survivors Compared with Only Age 50+ or Long-Term Survivors, HIV Aging and Long-Term Survivors Survey, 2021

| **Health Condition** | **Both Age 50+ and Long-Term Survivors** | **Either Age 50+ or Long-Term Survivors** | **Sig.** |
| --- | --- | --- | --- |
| Cancer Survivor | 23.5% | 4.7% | * |
| Diabetes | 12.3% | 2.3% | ^ |
| Heart Disease | 18.5% | 4.7% | * |
| High Cholesterol | 46.9% | 27.9% | * |
| History of Hepatitis | 28.4% | 9.3% | * |
| Hypertension/High blood pressure | 46.9% | 16.3% | ** |
| Lipodystrophy | 29.6% | 9.3% | ** |
| Overweight | 43.2% | 20.9% | ** |
| Taking Medications for Pain | 34.6% | 16.3% | * |

Note: ^ p<0.10, * p<0.05, ** p<0.01

Table S2. HIV+ Diagnoses by Region Through December 2020 and HIV Aging and Long-Term Survivors Survey in August 2021, New York State

| **Region** | **HIV+ in New York State** | | **HIV+ in Sample** | |
| --- | --- | --- | --- | --- |
|  | **Number** | **Percent** | **Number** | **Percent** |
| New York City | 82,756 | 78.4% | 53 | 46.1% |
| Manhattan | 21,253 | 20.1% | 26 | 22.6% |
| Bronx | 24,392 | 23.1% | 12 | 10.4% |
| Brooklyn | 22,080 | 20.9% | 9 | 7.8% |
| Queens | 13,128 | 12.4% | 3 | 2.6% |
| Staten Island | 1,903 | 1.8% | 3 | 2.6% |
| Long Island | 5,305 | 5.0% | 16 | 13.9% |
| Remainder of New York State | 17,549 | 16.6% | 46 | 40.0% |
| Capital District | 2,596 | 2.5% | 11 | 9.6% |
| Central New York | 1,441 | 1.4% | 3 | 2.6% |
| Finger Lakes | 2,751 | 2.6% | 14 | 12.2% |
| Mid-Hudson – Hudson Valley | 5,836 | 5.5% | 3 | 2.6% |
| Mohawk Valley | 644 | 0.6% | 2 | 1.7% |
| North Country | 450 | 0.4% | 1 | 0.9% |
| Southern Tier | 1028 | 1.0% | 3 | 2.6% |
| Western New York | 2803 | 2.7% | 9 | 7.8% |
| Total Sample | 105,610 | 100.0% | 115 | 100.0% |

Source: AIDS Institute (2021). New York State HIV/AIDS Annual Surveillance Report: For persons diagnosed through December 2020. Retrieved from May 26, 2022, https://www.health.ny.gov/diseases/aids/general/statistics/annual/2020/2020_annual_surveillance_report.pdf

**Section B. HIV and Aging and Long-Term Survivors 2021 Survey**

# Informed Consent

##
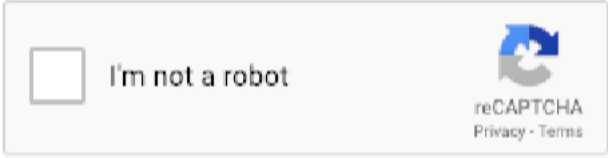
Please confirm that you are not a robot

My name is Maria Brown. I am a professor at Syracuse University’s School of Social Work.

**Purpose of the study**

I would like to learn about the healthcare experiences of long-term survivors of HIV/AIDS and people over 50 who are living with HIV/AIDS in New York State. I want to hear from consumers and providers across the state about barriers you have encountered related to HIV care and about recommendations you would make to the AIDS Institute to address these barriers and improve healthcare and supportive services.

I have consulted with the co-chairs of the HIV+ Aging and Long-Term Survivors Subcommittee on the focus and content of this survey, and will be sharing my results with them.

**Purpose of the study**

The HIV+ Aging and Long-Term Survivors Subcommittee wants to learn more about the healthcare experiences of long-term survivors of HIV/AIDS and people over 50 who are living with HIV/AIDS in New York State. We want to hear from consumers and providers across the state about barriers you have encountered related to HIV care and about recommendations you would make to the AIDS Institute to address these barriers and improve healthcare and supportive services.

**Procedures**

I am inviting you to participate in a research study by answering a web-based survey. This survey will take approximately 15-20 minutes of your time. Involvement in the study is voluntary. This means you have the right to refuse to take part in the study, without penalty. If you choose to take part and later change your mind, you have the right to withdraw from the study at any time, without penalty. Your choice to participate or withdraw from the study will not affect your relationship with the AIDS Institute or with your healthcare providers. They will not know if you participate in the study or see any of your responses to the survey.

All of your answers will be kept anonymous. This means that your name will not appear anywhere and your specific answers will not be linked to your name in any way. Your individual responses will not be seen by anyone other than the researcher. In the future, if we share data from this study in publications, presentations, or reports to the AIDS Institute, we will only include data for groups of people and not individuals.

At the end of the survey, you will be asked if you are willing to participate in an online town hall/focus group to explore barriers and recommendations reported in the survey. We will ask for an email address to send you an invitation to this town hall. That email address will not be linked to your survey responses, and we will make every effort to protect your anonymity.

**Internet Privacy**

Whenever one works with email or the internet, there is always the risk of compromising privacy, confidentiality, and/or anonymity. Your confidentiality will be maintained to the degree permitted by the technology being used. It is important for you to understand that no guarantees can be made regrading the interception of data sent via the internet by third parties.

**Instructions to complete the survey:**

To choose a response, click on the button or checkbox next to your answer. If you would like to change your answer, just click on a different button, or for questions with multiple answers just click in the checkboxes for all choices that are relevant to you. Click ‘NEXT’ to proceed to the next question. You may skip questions. After the entire survey has been completed please click on the 'SUBMIT' button on the last page. We appreciate your time and effort in assisting us by completing this web-based survey.

**Potential benefits to subjects and/or to society**

The benefit of this research is that you will be helping us to better understand the barriers encountered by older patients and long-term survivors living with HIV/AIDS in different regions and across the state. It will help the AIDS Institute identify ways in which care can be improved in clinics and in other service agencies that work with these patient populations.

**Potential risks or discomforts**

This study presents no more than minimal risk of harm to human subjects. Questions revolve around HIV healthcare and social experiences. None of the questions in this study are potentially dangerous, harmful, or embarrassing, and no deception of any kind has been used. However, it is always possible that a participant could become upset when answering questions. In this event, we have provided phone numbers to receive mental health support from the AIDS Institute here: General information line –

1-800-541-2437 English; 1-800-233-7432 Spanish

**Contact Information:**

**If you have any questions, concerns, complaints about the research, please contact the researcher Maria T. Brown at 315-443-4685 or** [**mbrown08@syr.edu.**](mailto:mbrown08@syr.edu) **If you have any questions about your rights as a research participant, you have questions, concerns, or complaints that you wish to address to someone other than the investigator, or if you cannot reach the investigator, contact the Syracuse University Institutional Review Board at** 315-443-3013**.**

I am 18 years of age or older.


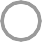
 Yes


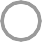
 No

I understand the procedures described above and I understand fully the rights of a potential subject in a research study involving people as subjects. My questions have been answered to my satisfaction. I have printed a copy of this consent form for my own records, and I consent to participate in this research study.

By clicking "I consent" below, I agree to participate in this research study and I confirm that I have not already responded to this survey.


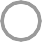
 I consent, begin the study


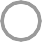
 I do not consent, I do not wish to participate

# Demographics

## Are you responding to this survey as a (choose the perspective most meaningful to you)?


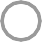
 Consumer/person living with HIV/AIDS


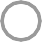
 Clinician


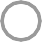
 Supportive services provider

## Do you practice in New York State?


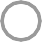
 Yes


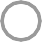
 No

## How many years have you been working with HIV+ patients?

Where is your practice located? (please select the option that best represents your practice)


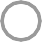
 City


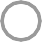
 Suburb


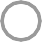
 Rural/Other

## Type of practice (please select the option that best represents your practice):


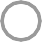
 Academic


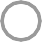
 Group practice


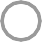
 Stand-alone

## Type of patients you serve


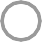
 People with HIV exclusively


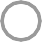
 General population

## Do you live in New York State?


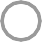
 Yes


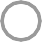
 No

## Which of these terms describes you? (Select all that apply)


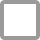
 50 years of age or older


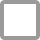
 Long-term HIV survivor


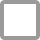
 Neither

## How long have you been diagnosed with HIV?

Do you feel like you have someone you can trust with knowledge of your HIV status?


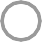
 Yes


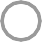
 No

## Age

Where do you live?


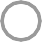
 City


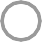
 Suburb


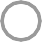
 Rural/Other

## Sex assigned at birth:


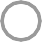
 Male


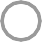
 Female

## Gender identity:


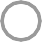
 Male


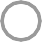
 Female


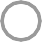
 Gender non-conforming


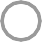
 Transgender FTM


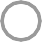
 Transgender MTF


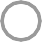
 Transgender non-binary


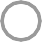
 Other (please specify)

## How do you self-identify? Please check all that apply:


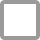
 White or Caucasian


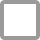
 Black, African American, or Afro-Caribbean


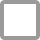
 Hispanic or Latinx origin


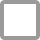
 South Asian (Indian, Pakistani, Bangladeshi, or other South Asian)


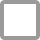
 East Asian (Chinese, Japanese, Korean, or other East Asian)


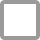
 Southeast Asian (Vietnamese, Filipino, Cambodian, or other Southeast Asian)


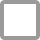
 Middle Eastern or North African


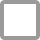
 American Indian or Alaska Native


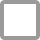
 Pacific Islander or Native Hawaiian


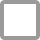
 Unknown


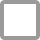
 Other race or origin (please specify):

## Do you speak a language other than English at home?


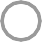
 Yes


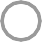
 No

## How well do you speak English?


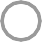
 Very well
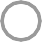
 Well


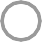
 Not well


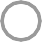
 Not at all

## Relationship status:


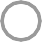
 Married


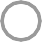
 Living with partner


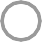
 Divorced or separated


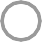
 Single


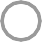
 Widowed


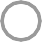
 Prefer not to say


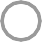
 Other (please specify):

## Living arrangement


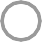
 Living with spouse/partner


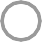
 Living with family


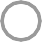
 Living with non-family


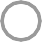
 Group housing


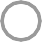
 Living alone


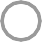
 Temporarily housed


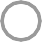
 Unstably housed or homeless

# General Health Information

## Now I would like to ask you about any health concerns you may be having.

Which of the following health conditions do you have? (Check all that apply)


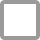
 Alcohol or substance abuse disorder


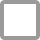
 Alzheimer's disease or other dementia


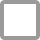
 Anemia


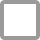
 Anxiety


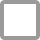
 Asthma


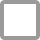
 Cancer survivor


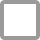
 Currently in cancer treatment


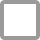
 Depression


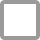
 Diabetes


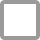
 Heart disease


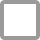
 High cholesterol


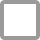
 History of hepatitis


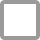
 Hypertension / high blood pressure


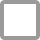
 Kidney disease


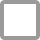
 Lipodystrophy


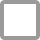
 Lung disease, COPD, emphysema, or chronic bronchitis


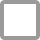
 Osteoporosis or low bone density


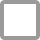
 Overweight


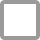
 PTSD


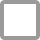
 Peripheral neuropathy


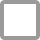
 Prediabetes or borderline diabetes


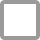
 Underweight


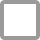
 Other (please specify):


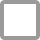
 None

## Have you been having any of the following problems? (Check all that apply)


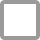
 I have fallen in the past 6 months


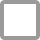
 I use a cane or walker


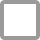
 I use a wheelchair


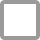
 Problems with hearing


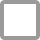
 Problems with vision


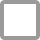
 I take medications for pain


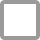
 Other (please specify):


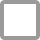
 None

## Would you say your health in general is excellent, very good, good, fair, or poor?

Excellent

Good

Average

Poor

Terrible

# Barriers and Recommendations

In August of 2020, the HIV+ Aging and Long-Term Survivors Subcommittee hosted a series of online town halls that consisted of long-term survivors and older people with HIV and clinicians who met in small groups to *identify barriers* and *make recommendations* to address these barriers. The categories identified by participants ranged across healthcare services and supportive services.

1. Clinical Care and Coordination
2. Telemedicine/Telehealth
3. Medication and Pharmacy Services
4. Aging and Long-Term Care
5. Mental Health and Social Support
6. Barriers During COVID-19
7. Health Equity and Stigma
8. Transportation and Food
9. Housing
10. Financial Support

We would like you to identify the 3 most important *barriers* and 3 most important *recommendations* in each of these categories.

You will have an option to enter an additional barrier or recommendation as one of your 3 choices in each category.

Clinical Care and Coordination

Please select the *3 most important barriers* that you are facing in this area.

Not enough HIV primary and specialty care providers in upstate NY and rural areas

Being on Medicaid limits access to appropriately trained providers, especially in specialty care

Lack of care coordination across different departments and specialties

Clinicians are not asking PLWHA about substance use or sexual health

Lack of support to help PLWHA manage care

Other (please specify 1 additional barrier):

None

Please select the *3 most important recommendations* you would make in this area.

Increase funding for clinics and facilities in underserved and understaffed areas

Integrate HIV care with other specialties so PLSHA have different needs met in one place

Use electronic medical records for communication between staff and between clients and care teams

Provide clinicians with relevant experience to help older PLWHA and LTS manage their complex care needs

Certified peer workers (CPWs) should be included in initial visit with provider and also assist PLWHAs in managing medications, etc.

Other (please specify 1 additional recommendation):

None

## Telemedicine/Telehealth

Please select the *3 most important barriers* that you are facing in this area.

Lack of knowledge or comfort with evolving technology

PLWHA cannot afford to access technology (i.e. phone plan, internet plan, Wi-Fi, etc.)

Lack of quiet and safe private space to have telehealth visit

Telehealth makes it harder for PLWHA (especially newly-diagnosed) to discuss their medical issues and connect with their clinician

PLWHA may find it easier to avoid appointments or not take medications when using telehealth

Other (please specify 1 additional barrier):

None

Please select the *3 most important recommendations* you would make in this area.

CPWs should be included in initial telehealth visit with provider ad also assist PLWHAs in using technology

Provide subsidies so PLWHA can afford current technologies for telehealth

Pay attention to the psychological impact of transitioning to or starting care with telehealth

Mandate annual in-person visits to ensure that PLWHA's needs are being met

Other (please specify 1 additional recommendation):

None

## Medication and Pharmacy Services

Please select the *3 most important barriers* that you are facing in this area.

Lack of sensitivity training for pharmacy staff which might lead to status disclosure and stigma

Pharmacists need training to understand HIV from the consumer's point of view

Up to pharmacists to catch potential drug interactions when the primary care doctor is not the sole prescriber of medication

Medication delivery services are not always reliable or available at all pharmacies especially during the COVID-19 pandemic

Other (please specify 1 additional barrier):

None

Please select the *3 most important recommendations* you would make in this area.

More education and trainings for pharmacy staff on cultural sensitivity, confidentiality, and insurance coverage

Give PLWHA a day or two of medications before leaving the ER

Advertise AIDS Institute HIV medication assistance telephone number (and push to include ALL medications an HIV+ person is currently taking)

Advocate for pharmacies to fill prescriptions within 24 hours and stay open longer to fill prescriptions

Create a delivery service for medications, like UberEATS

Other (please specify 1 additional recommendation):

None

## Aging and Long Term Care

Please select the *3 most important barriers* that you are facing in this area.

Lack of home health care options and long-term care facilities in rural areas

Lack of geriatric providers on staff at HIV clinics

Long-term care facilities are not equipped to take care of PLWHA and sometimes reject them because of expensive HIV medications

PLWHA face stigma and homophobia in assisted living facilities

Ageism towards older people and younger people who are long-term survivors

Long-term care facilities do not always provide a cane, walker, or wheelchair and may not let you bring your own

PLWHA have multiple health conditions and aging-related complications occurring with HIV that are not acknowledged or addressed

Perinatally-infected PLWHA who were born between the 80s and 90s are often forgotten in discussion of long-term survivorship

End-of-life planning doesn't happen or it is avoided until the last minute

Other (please specify 1 additional barrier):

None

Please select the *3 most important recommendations* you would make in this area.

Expand long-term care facilities through satellite locations to rural areas

Provide assisted living and non-assisted living options for older PLWHA

Standardize infrastructure and policies so long-term care facilities can provide better services for PLWHA and people with mobility challenges

Educate PLWHA and providers on the effect of HIV/AIDS on aging and comorbidities for PLWHA of different ages

Educate PLWHA about the management of pain and other symptoms

Providers should screen PLWHA for aging-related symptoms when they reach 50 years of age

Aging PLWHA and long-term survivors need better care coordination between providers, social workers, and mental health services

Use CPWs to support aging PLWHA and provide resources on long-term survivorship and growing older with HIV

Begin conversations about end-of-life planning sooner rather than later

Spirituality is related to aging and culture - ask PLWHA what they want

Offer mental health services for PLWHA who are making end-of-life decisions

Other (please specify 1 additional recommendation):

None

## Mental Health and Social Support

Please select the *3 most important barriers* that you are facing in this area.

Not all clinics provide access to mental health services

Lack of access to self-care services, alternative therapies, and wellness centers

Clinicians are not asking about mental health

Lack of therapists/counselors that are representative of clients (share the same race, gender, age, or cultural background)

Not enough coverage provided for mental health through Medicaid or other insurance plans

Other (please specify 1 additional barrier):

None

Please select the *3 most important recommendations* you would make in this area.

Make crisis hotline information and mental health resources easily accessible for PLWHA

Providers should routinely ask PLWHA about their mental health

More patient education on importance of self-care and mental health therapy particularly for Hispanic community

Hire counselors and mental health professionals that represent communities they service

Counselors need to treat PLWHA as individuals and meet clients where they are (physically and mentally)

Mental health programs that are multilingual, culturally inclusive, and culturally sensitive

Other (please specify 1 additional recommendation):

None

## Barriers During COVID-19

Please select the *3 most important barriers* that you are facing in this area.

Not enough mental health providers to help PLWHA cope with the added trauma of COVID-19, such as isolation and depression

Not being able to see people or attend support groups in person due to COVID-19

During the pandemic, it is easy to isolate, drop out of care, and not be held accountable

It is difficult to use or access technology to remain connected with other people

Having to work during the pandemic is very stressful and exhausting

Other (please specify 1 additional barrier):

None

Please select the *3 most important recommendations* you would make in this area.

Make one-on-one counseling widely available through telehealth during and after COVID-19

Notify PLWHA when in-person appointments are available, rather than leaving the burden on PLWHA to keep checking

CPWs should make regular check-in calls to PLWHA who are isolated during COVID-19

Provide support for community-organized socially distanced activities and electronic support groups (e.g. via Zoom and other safe social media)

Other (please specify 1 additional recommendation):

None

## Health Equity and Stigma

Please select the *3 most important barriers* that you are facing in this area.

PLWHA face stigma in both HIV and non-HIV specialties (i.e. dentistry) based on their race, immigration status, sex work, and/or substance use

Minority PWLHA do not trust the healthcare system because of historic racism and trauma

Lack of clinical HIV research done with women, older adults, and heterosexual people

Lack of training on trauma-informed care, cultural sensitivity, and HIV services for all healthcare staff

Lack of communication services for hearing impaired or deaf PLWHA and staff knowledge of how to properly use services

Lack of multilingual translation services and staff knowledge of how to properly use services

Lack of access to education and educational materials that are easy to read and are in multiple languages

Lack of healthcare providers that PLWHA can identify with or relate to

PLWHA may not know how to ask their clinician/provider questions to help them understand their health and healthcare

Lack of informal or formal process for people experiencing stigma to talk to someone or file a complaining

Other (please specify 1 additional barrier):

None

Please select the *3 most important recommendations* you would make in this area.

Community partnerships should provide more regular training for all healthcare staff on trauma-informed care, cultural competency, and HIV services

Diverse PLWHA (age, modes of transmission, etc.) and demographic representation among community boards, clinic staff, and supportive services staff that PLWHA can identify with

Appropriate services for hearing impaired or deaf people as a standard of care

More translation services, more multilingual staff and CPWs, phone numbers and hotlines in multiple languages, and all referrals should include statement of consumer's preferred language

Provide educational opportunities and materials that are easier to read and understand in order to improve PLWHA health literacy

Have CPWs on staff to help PLWHA communicate with their doctor and ask questions

More PLWHA input on all quality improvement activities such as clinical practices and staff trainings

Provide access to trained staff to handle complaints about facing stigma in healthcare settings

Expand positive messaging on LGBTQ+ history and sexual health education

HIV awareness campaigns should be inclusive of all races, ethnicities, ages, sexual orientations, lifestyles, etc.

Other (please specify 1 additional recommendation):

None

## Transportation and Food

Please select the *3 most important barriers* that you are facing in this area.

Lack of public transportation (e.g. buses, taxis, subways, etc.) that is affordable, available, and accessible to all no matter their age, income, or insurance status

Living too far away or out of route for transportation services

Ryan White transportation issues increase as funding decreases, especially for aging population

Pantries have limits and may not have food for people with specific nutritional needs

It is difficult for people living alone who can't cook on their own to access healthy foods

Food desserts have inadequate bus services that make it difficult to reach faraway supermarkets

Other (please specify 1 additional barrier):

None

Please select the *3 most important recommendations* you would make in this area.

Provide more affordable and accessible transportation options for aging and mobility-challenged PLWHA

Providers should connect PLWHA with a Registered Dietician or Certified Nutritionist who can provide PLWHA with ideas on how to prepare healthier foods including cultural favorites

Use CPWs to educate PLWHA on resources including food vouchers and local food banks

Organize state-funded and volunteer-led food banks that provide nutritional food at affordable prices

Enable food drives, programs, and pantries to provide more services each month to PLWHA

Provide food delivery options for aging and mobility-challenged PLWHA

Patient assessments should include questions about food security and transportation

Other (please specify 1 additional recommendation):

None

## Housing

Please select the *3 most important barriers* that you are facing in this area.

Lack of safe, accessible, and affordable senior housing

Lack of attention and care for homeless population living with HIV

Barriers to housing based on age and eligibility

There are long waiting lists for housing

Changes in housing including rent prices and late fees during COVID-19

Other (please specify 1 additional barrier):

None

Please select the *3 most important recommendations* you would make in this area.

Build more housing and make more housing available to low income populations

Make homelessness agencies aware of HIV-related needs

Agencies that receive housing assistance funds should include consumer input and clarify their definitions of affordable housing

Patient assessments should include questions about housing

Other (please specify 1 additional recommendation):

None

## Financial Support

Please select the *3 most important barriers* that you are facing in this area.

Enhanced unemployment benefits due to COVID-19 have ended

PLWHA do not know enough about Social Security and Disability (SSI and SSDI) benefits

Any increase in income reduces SSI/SSDI benefits and coverage for Medicaid, copays, medical visits, housing benefits

Even with insurance, copays are too high for emergency or urgent care

PLWHA need unbiased assistance to help them apply for high quality health insurance

Healthcare coverage can be interrupted when insurance requirements change

Insurance companies will switch to cheaper meds without consulting PWLHA

Since COVID-19, there are conflicting messages about Medicaid recertification or changes to benefits

Other (please specify 1 additional barrier):

None

Please select the *3 most important recommendations* you would make in this area.

More funded programs to help PLWHA pay for housing, food, and transportation

More leeway/unlimited extra income for PLWHA over 50 who have SSI/SSDI

Better advertising of services that help PLWHA obtain high quality health insurance or help pay for medications (for example, ADAP)

Revise patient rights so insurance companies cannot be changed or interrupted without consulting the consumer and clinician

More funding for mental health services/organizations accepting Medicaid

Provide clear and consistent messaging about recertification to changes to benefits (Medicaid, rental assistance, food stamps, etc.)

Pay certified peer workers (CPWs) a living wage

Other (please specify 1 additional recommendation):

None

# Conclusion

Following Up On This Survey:

We will be conducting at least one online town hall/focus group to explore barriers and recommendations reported in this survey. If you are interested in taking part in that discussion, please provide your email below. This email address will not be linked to our responses to the survey, or shared with anyone else who takes part in the town hall/focus group, and we will make every effort to protect your anonymity.

If you do not want to be contacted, click Next.

If you do wish to be contacted for follow-up, enter your email address here:

Powered by Qualtrics
